# Pharmacokinetics and thrombolytic effects of the recombinant tissue-type plasminogen activator in horses

**DOI:** 10.1186/1746-6148-9-158

**Published:** 2013-08-09

**Authors:** Wolfgang Bäumer, Gudrun M Herrling, Karsten Feige

**Affiliations:** 1 Department of Pharmacology, Toxicology and Pharmacy, University of Veterinary Medicine Hannover, Foundation, Buenteweg 17, 30559 Hannover, Germany; 2 Department of Molecular Biomedical Sciences, College of Veterinary Medicine, North Carolina State University, Raleigh, USA; 3 Clinic for Horses, University of Veterinary Medicine Hannover, Foundation, Buenteweg 17, 30559 Hannover, Germany

**Keywords:** Alteplase, Recombinant tissue plasminogen activator, Thrombus, Clot formation, Hypercoagulopathy

## Abstract

**Background:**

To test the efficacy of the recombinant tissue-type plasminogen activator (rt-PA) alteplase in horses, the thrombolytic effect was tested in *in vitro* generated equine thrombi. The extent of lysis was determined by measuring the decrease in thrombi weight over a period of 4 hours. *In vivo* pharmacokinetics of alteplase were determined in 6 healthy horses. A single dose (1 mg/kg) was applied via intravenous infusion over a period of 30 minutes Coagulation-related variables, blood count and clinical parameters were taken before the treatment and until 48 h after treatment. In addition, plasma rt-PA concentration was measured until 300 min after commencing the infusion.

**Results:**

*In vitro*, a dose dependent decrease of thrombus weight ranging from a 56 (± 6.5) % decrease for 0.5 μg/ml to 92 (± 2.1) % decrease for 5 μg/ml rt-PA was noted. The D-dimer concentration in the lysis medium correspondingly increased from 0.10 up to 10.8 mg/l.

*In vivo*, none of the horses showed an adverse reaction to the alteplase infusion. In some horses blood parameters were slightly altered. The 1 mg/kg dose yielded the following pharmacokinetic parameters: Cmax = 1.25 ± 0.27 μg/ml; CL = 21.46 ± 5.67 ml/min/kg; dominant half life (t1/2α) = 6.81 ± 1.48 minutes; median elimination half life (t1/2β) = 171 min (range: 85–1061); AUC = 50.33 ± 17.62 μg · min /ml.

**Conclusion:**

These findings indicate that a single dose of 1 mg/kg alteplase results in rt-PA plasma concentrations comparable to those in humans and might be sufficient for a thrombolytic therapy in horses. Further studies must be performed to determine the alteplase effectiveness in horses with jugular vein thrombosis.

## Background

Severe systemic diseases such as infections, inflammatory disorders, septicemia, endotoxemia, and colic can be associated with an activated coagulation state and predispose horses to develop thrombophlebitis or thrombosis. Risk of clot formation at the site of venepuncture is enhanced by frequent venepuncture of the jugular vein, or the administration of irritant pharmaceuticals, or by insertion of catheter systems [1,2]. Treatment of thrombosis is directed against further thrombus formation and commonly performed with unfractioned heparin [3] or low-molecular weight heparin [4]. Although recombinant hirudin, a specific thrombin inhibitor that dissolves thrombi, has been investigated experimentally [5], thrombolytic agents are not commonly employed in equine medicine.

Tissue-type plasminogen activator (t-PA) is an endogenous enzyme that induces lysis of intravascular thrombi by the activation of plasminogen to plasmin, which degrades the fibrin components of blood clots [6]. Alteplase is a recombinant human tissue-type plasminogen activator. It is frequently used as a thrombolytic agent in human patients with cardiac infarction or stroke [7,8]. In veterinary medicine, experience with rt-PA is limited [9] and derive mainly from experiences with cats suffering from aortic thromboembolism and case reports in dogs [9,10]. There are also few case reports for the use of rt-PA in horses. In one case report a foal with aorto-iliac thrombosis received a 2 mg dose of rt-PA intravenously [11]. Recombinant t-PA was also used locally to treat a fibrinous pericarditis in a horse [12]. An intrapleural instillation of alteplase and the newer generation plasminogen activator tenectoplase was used in a fibrinolytic treatment of septic pleuropneumonia [13,14]. Recently, enecteplase was also used locally to treat hyphema in a horse after ocular trauma [15]. However, to the author’s knowledge, pharmacokinetic data of rt-PA in horses have not been reported yet.

Combined with commonly used anticoagulants, for example heparin, rt-PA extends/augments thrombolytic therapy options for humans and potentially for animals as well [6,16]. Maximum plasma concentrations in humans during the infusion (C_max_) of 0.5 mg/kg t-PA are in the range of 900 to 1300 ng/ml [6,16]. These concentrations were taken as approximate target values for the *in vivo* study in horses. Thus, in the present study, the *in vitro* capability of rt-PA on equine thrombi was tested and the pharmacokinetics were evaluated *in vivo* in 6 adult horses.

## Methods

### *In vitro* study

Blood was collected from six mares and one gelding (all Hannoverian horses) aged between 4 and 25 years. Horses were owned by the Clinic for Horses, University of Veterinary Medicine, Hannover. All horses were clinically healthy and haematological variables (WBC, RBC, haematocrit, thrombocytes, fibrinogen, total plasma protein) were within normal ranges. They had no systemic diseases and were not involved in research projects 4 weeks prior to the study. The horses were kept in individual box stalls, fed hay and grain and were on pasture daily. The animal experiment was approved by the German Government (LAVES, no. 09/1796). Blood was obtained by venepuncture of the jugular vein and collected into 50 ml vials containing sodium citrate (1.3%) to prevent clotting. Subsequently, blood was centrifuged (3000 g, 4°C, 10 min) and plasma was stored at 4°C until used.

Siliconized glass vials (24 ml, Fa. Schott Fiolax, Mainz, Germany) were filled with 4250 μl 0.9% NaCl, 200 μl Ca-thromboplastin solution (Thromborel S®, Fa. Dade Behring, Chicago, USA), and 500 μl CaCl_2_-solution (1.3%) and incubated at 37°C for 20 min. Sodium citrate blood (1000 μl) was added, and the vials were sealed with parafilm (Parafilm M®, Firma Brand GmbH und Co AG, Wertheim, Germany) and inverted several times. For coagulation the vials remained at 37°C for four hours. The thrombi had a mean (± SD) weight of 2.92 ± 0.31 g. From each horse 6 to 20 thrombi were generated. The thrombi were transferred to Petri dishes (diameter 10 cm) and the thrombi were fixed with a cotton wire.

Thrombolysis was performed in a water bath pre-warmed to 37°C. Glass insets contained 20 ml autologous horse plasma. Thrombi fixed with cotton fibres were submerged in plasma. Plasma was continuously stirred by means of a magnetic stirrer (150 U/min). Effects of rt-PA on equine thrombi were tested at concentrations of 0.5, 1.5, 3.5 and 5 μg/ml. Four horses were used for the complete dose range (0 to 5 μg/ml rt-PA) and three additional horses were taken for 0 and 1.5 μg/ml rt-PA. For each concentration three to four thrombi obtained from each horse were tested. For each time point and concentration the average of these three to four thrombi was taken.

Thrombolysis was determined by weight loss. Every hour thrombi were transferred to a glass beaker filled with 15 ml 0.9% NaCl and weighed. For the determination of the fibrin degradation product D-dimer, 200 μl of the supernatant was collected every hour and stored at −20°C until determination. D-dimer was detected with a reflectometer (NycoCard® Reader II, Axis-Shield PoC AS, Oslo, Norway) according to the manufacturer’s protocol with in house- validation for horse samples.

### *In vivo* study

Four mares and two geldings (three trotters, two Hannoverian horses, one Swiss crossbred) were included in the main *in vivo* study (no horses of the *in vitro* study were used for the *in vivo* study). They were 5 to 17 years old and weighed between 488 and 680 kg. The study was approved by the German Government (LAVESno. 09/1796).

Two horses were used for dose finding studies and 6 horses were used for pharmacokinetic data analysis. The general health status (heart frequency, respiratory rate, evaluation of mucosa) was monitored frequently at the beginning of rt-PA infusion and the frequency was reduced over the 24 h observation period.

The horses received catheter systems (EquiCath™ Fastflow; 12 G; 450 ml/min; B. Braun Vet Care GmbH, Tuttlingen, Germany) in the right and left jugular veins. One for administration of alteplase and one for blood sampling. Before infusion of alteplase samples for hematological examination were collected (t = 0) into different vacutainers (Vacuette® 4 ml, K3EDTA, Greiner Bio- One GmbH, Frickenhausen, Germany). After collection, blood samples were centrifuged (10 min, 4°C, 3000 g) and plasma was stored at −80°C until analysis. Additionally, coagulation status was checked every hour after infusion started.

The rt-PA alteplase (Actilyse®, Boehringer-Ingelheim Pharma GmbH und Co. KG, Ingelheim am Rhein, Germany) was freshly prepared by dissolving the dry substance with the appropriate accompanying solvent to a final concentration of 2 mg/ml. Alteplase was administered intravenously over a 30 min period by means of an infusion pump (Infusomat® fmS, B. Braun, Melsungen, Germany). In a pilot study one horse received 0.25 mg/kg and another received 0.5 mg/kg. In the main study, six horses received 1 mg/kg alteplase. Blood samples were taken directly before infusion (0), and 1, 6, 12, 18, 24, 30, 36, 48, 60, 72, 84, 96, 108, 120, 135, 150, 165, 180, 195, 210, 225, 240 and 300 min after infusion of alteplase. Samples were collected into a vacutainer (Vacuette® 9 ml 9NC Coagulation sodium citrate 3.2%, Greiner Bio-One GmbH, Frickenhausen, Germany). The first 10 ml of blood obtained were discarded and the following 10 ml were collected. The permanent-catheter systems were immediately flushed with 10 ml 0.9% NaCl solution following insertion.

#### Analysis of hemostasis and rt-PA in plasma

Activated partial thromboplastin time (APTT-S TEClot-Reagens, TECO Medical instruments Products and Trading GmbH, Neufahrn, Germany and calcium-chloride-solution (0,025 mol/l) Siemens Healthcare Diagnostics Products GmbH, Marburg, Germany), the prothrombin time (Thromborel® S, Siemens Healthcare diagnostics products GmbH, Marburg, Germany), the thrombin time, TT (Test Thrombin Reagens, Siemens Healthcare Diagnostics Products GmbH, Germany) and fibrinogen (Multifibrin U Siemens Healthcare Diagnostics Products GmbH, Germany) were determined by means of a coagulometer (KC 1A, Amelung, Lemgo, Germany).

Hematocrit, erythrocyte count, leukocyte count and thrombocyte count were determined on an automated analyser (Sysmex KX-21 Deutschland GmbH, Norderstedt, Germany). Agglutination of thrombocytes was excluded microscopically. Total protein was analysed by means of a refractometer (Euromex, Arnheim, Netherlands).

The determination of rt-PA was performed via enzyme linked immunosorbant assay (ELISA) (AssayMax Human Tissue-Type Plasminogen Activator ELISA Kit, Assay Pro, St. Charles, MO, USA). The standard calibration was carried out in horse plasma diluted 1:10 with dilution media, as the instructions of the test kit required dilution of at least 1:10 to minimize plasma matrix interactions with the ELISA. The calibration with rt-PA used for the study and with the rt-PA provided with the kit were both highly linear between 0.03 and 1 ng/ml, when plasma was diluted to at least 1:10. Calibration curves with higher concentrations of horse plasma led to non-linear curves. All samples were thus diluted to at least 1:10 and plasma samples were diluted after pilot measurements to fit the linear area of 0.03 and 1 ng/ml. The intra-assay variance was 6.2%, inter-assay variance, however, was up to 48.2%. Inter-assay variance might be due to repeated freeze-thaw cycles of the samples during the validation. It was therefore decided to aliquot the samples of the main experiment to avoid repeated freeze-thaw cycles. Additionally, crucial time points (around C_max_) were measured on two different ELISAs and and the mean of the two determinations was taken for analysis.

#### Statistical analysis

The results were analysed to determine whether there was a significant difference between thrombus weights after four hours of incubation. Analysis was performed by one way ANOVA followed by an adjusted Dunnett’s post hoc test for unequal sample sizes. Changes in haematological parameters over time were analysed by t-tests for multiple comparisons using Bonferroni’s adjustment.

The pharmacokinetic parameters were calculated using a two compartment model (WinNonlin 5.2, Pharssight Corp., Mountain View, CA, USA). The following pharmacokinetic parameters were calculated: Plasma concentration at the end of infusion (C_max_), area under the curve (AUC), total plasma clearance (Cl) and plasma half lives (t_1/2α_, t_1/2β_).

## Results

### *In vitro* study

**Figure 1:**
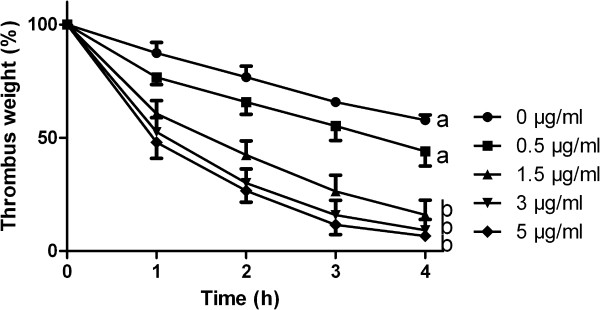
**Reduction in thrombus weight of *in vitro *generated equine thrombi after incubation with rt-PA (n = 4 horses for concentrations 0.5,3.0 and 5.0** **μg/ml**, **n = 7 for 0 and 1**.**5** **μg/ml rt-PA;all thrombi for each time point and concentration were performed a minimum of three times).** After four hours, there is a significant difference between 0 as well as 0.5 μg/ml and 1.5 μg/ml, whereas no significant difference was found between 3 as well as 5 μg/ml and 1.5 μg/ml, a vs. b: p < 0.001.

To determine the thrombolysis, the weight of the thrombi and generation of D-dimer were measured every 60 min over a period of four hours. There was a time dependent decrease in thrombus weight and an increase in D-dimer generation for equine thrombi (Figure 1, Table 1). An addition of 1.5 μg/ml rt-PA to the autologous horse plasma led to nearly 90% weight reduction of thrombi 4 hours after incubation, whereas 3.5 μg/ml and 5 μg/ml increased this thrombolytic activity only marginally.

**Table 1:** **D**-**dimer formation during thromboylsis of*in vitro*generated equine thrombi (n** = **4 horses for 0**.**5**, **3**.**5 and 5** μ**g/ml rt**-**PA**, **n = 7 for 0 and 1**.**5** μ**g/ml rt**-**PA**, **all thrombi for each time point and concentration were performed at least in triplicates)**

**t-PA (μg/ml)**	**0 h**	**1 h**	**2 h**	**3 h**	**4 h**
0	0.10 (± 0.00)	0.10 (± 0.01)	0.10 (± 0.01)	0.11 (± 0.04)	0.11 (± 0.03)
0.5	0.12 (± 0.03)	1.83 (± 0.76)	4.40 (± 1.49)	7.72 (± 3.28)	10.08 (± 3.70)
1.5	0.11 (± 0.03)	4.74 (± 2.55)	8.08 (± 4.97)	9.25 (± 4.35)	9.78 (± 4.87)
3.5	0.12 (± 0.03)	1.87 (± 0.74)	2.70 (± 0.99)	4.14 (± 1.97)	4.64 (± 2.22)
5	0.12 (± 0.03)	1.77 (± 0.55)	2.98 (± 0.84)	4.22 (± 1.67)	5.32 (± 2.44)

There was a time dependent increase of fibrin degradation product D-dimer by incubation with rt-PA, whereas no such increase was observed with control thrombi (no rt-PA added to horse plasma). The 0.5 and 1.5 μg/ml concentration led to highest D-dimer formation, whereas the effect was less pronounced at the higher concentrations (3.5 and 5 μg/ml rt-PA, Table 1).

### *In vivo* study

**Figure 2:**
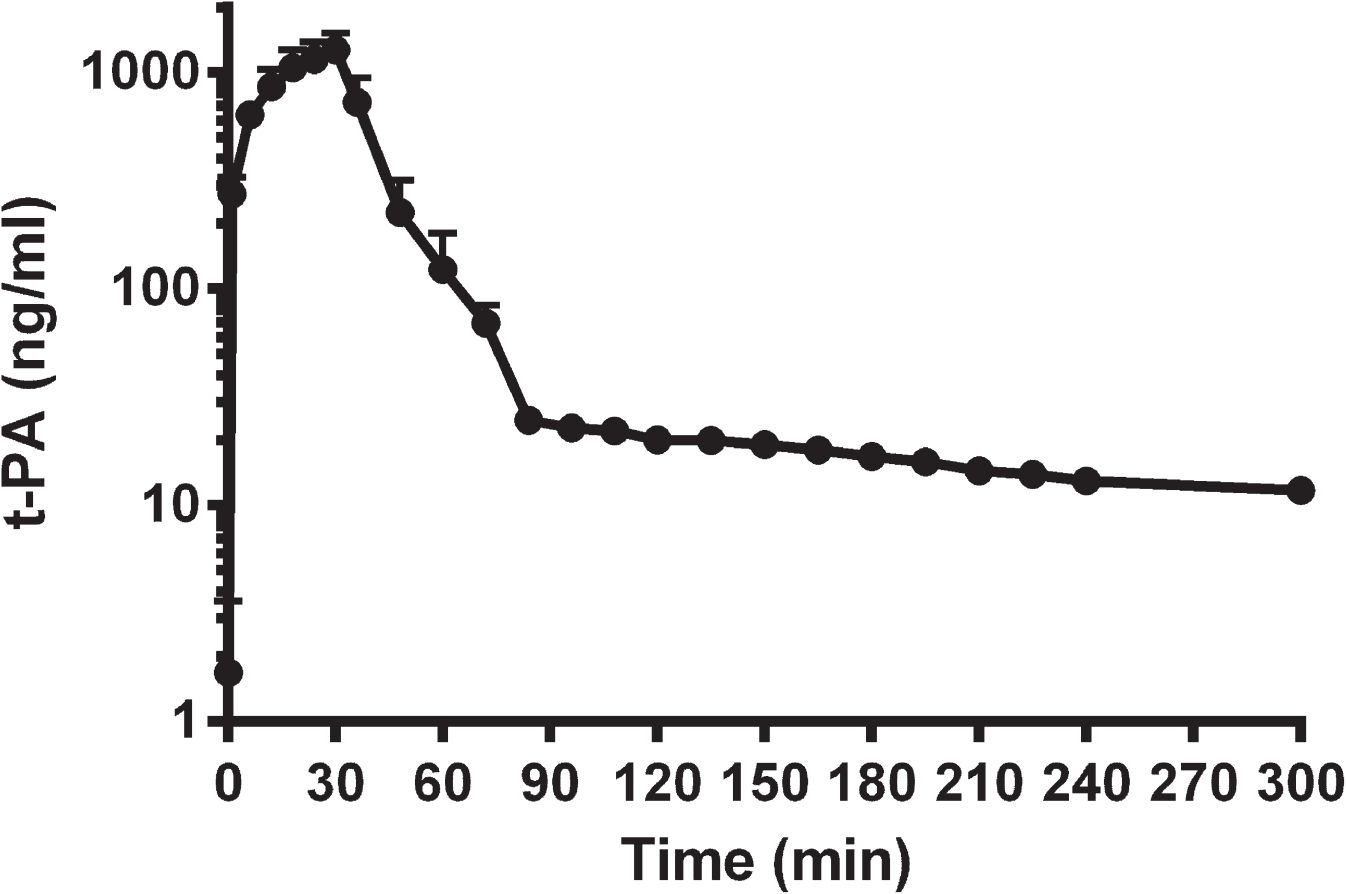
**Time curve of rt-PA plasma concentration (mean ± SD) after intravenous infusion of 1 mg/kg rt-PA over a period of 30 min in six healthy horses.**

Infusion of rt-PA alteplase was well tolerated in the different dosages by every horse. None of the horses showed adverse drug reactions. In a pilot study, one horse received a dose of 0.25 mg/kg alteplase. The maximal plasma concentration was 199 ng rt-PA/ml. By administration of 0.5 mg/kg rt-PA C_max_ was 779 ng/ml. In order to achieve plasma rt-PA concentrations consistent with those demonstrated to be thrombolytic *in vitro* and in line with target concentrations in humans (C_max_ around 1000 ng/ml) a dose of 1 mg/kg was used for the main experiment. When administered over 30 min via infusion, the mean maximal plasma concentration measured was 1252 (± 267) ng/ml rt-PA (Figure 2). The plasma clearance was 21.46 (± 5.67) ml/min/kg and t_1/2α_ was 6.81 (± 1.48) min followed by a delayed β phase (t_1/2β_ 317.93 ± 373.77 min). The high standard deviation is due to the extremely prolonged t_1/2β_ in one horse (1061 min). The median t_1/2β_ was 171 minutes with a range of 85–1061 min.

The AUC was quite high due to the prolonged t_1/2β_ (50.33 ± 17.62 μg•min/ml). Mean blood and coagulation parameters were within the reference range (Table 2). However, activated partial thromboplastin time at hour 4 observation time was significantly reduced compared to the basal value.

**Table 2:** **Effects of rt**-**PA on haemostasis and further blood parameters (reference values in parentheses) in 6 horses of the main experiment (mean** ± **SD)**, *significantly different from basal value (p < **0**.**05)**

**Time (h)**	**Thrombin time (sec; 17–21)**	**Prothrombin time (%; 70–100)**	**Partial thromboplastin time (sec; 43–63)**	**Fibrinogen (g/l; 1.0-1.2)**	**Thrombocytes (10**^**3**^**/μl; 90–300)**	**White blood cells (G/l; 5–10)**	**Red blood cells (T/l; 6.5-9.0)**	**Haematocrit (%; 32–45)**
0	18.67 (±1.97)	85.50 (±15.23)	55.67 (±7.94)	1.13 (±0.16)	120.50 (±44.60)	7.67 (±1.06)	7.31 (±0.99)	36.83 (±3.19)
1	18.17 (±1.47)	93.50 (±10.46)	51.67 (±5.96)	1.12 (±0.13)	132.50 (±37.18)	7.45 (±1.23)	7.64 (±0.48)	37.50 (±3.15)
2	17.17 (±1.33)	97.00 (±4.69)	47.50 (±8.04)	1.14 (±0.19)	126.00 (±37.46)	7.32 (±1.23)	7.59 (±0.51)	37.00 (±2.83)
3	18.17 (±1.72)	88.83 (±15.69)	48.83 (±5.74)	1.13 (±0.14)	128.50 (±34.05)	8.00 (±0.79)	8.04 (±0.85)	40.15 (±5.02)
4	17.67 (±1.37)	95.00 (±7.75)	45.17 (±6.40)*	1.13 (±0.17)	126.50 (±32.02)	7.75 (±1.07)	7.74 (±0.50)	38.63 (±2.94)
5	17.50 (±1.64)	90.33 (±14.72)	50.17 (±8.77)	1.09 (±0.13)	114.00 (±23.82)	7.95 (±1.38)	7.85 (±0.52)	39.17 (±3.54)
12	19.67 (±1.51)	89.00 (±13.77)	55.00 (±6.16)	1.08 (±0.11)	150.17 (±41.73)	7.67 (±1.50)	7.95 (±0.45)	39.30 (±2.07)
24	18.50 (±0.84)	89.33 (±16.54)	54.83 (±5.19)	1.10 (±0.16)	127.83 (±44.20)	8.02 (±1.19)	8.22 (±1.10)	41.33 (±4.46)
36	18.50 (±0.84)	87.17 (±20.77)	57.17 (±6.21)	1.15 (±0.14)	139.33 (±37.89)	7.73 (±1.57)	8.54 (±1.03)	41.33 (±3.44)
48	18.50 (±1.05)	85.17 (±20.22)	56.33 (±6.77)	1.13 (±0.12)	132.67 (±36.66)	7.62 (±1.06)	8.01 (±0.66)	38.73 (±3.44)

## Discussion

The aim of the present study was to characterize the human recombinant plasminogen activator alteplase (rt-PA) for use as a possible thrombolytic agent in equine patients suffering from thrombosis. After determining the *in vitro* thrombolytic activity in equine thrombi, pilot pharmacokinetic data were obtained in healthy horses. Present data indicate a dose dependent decrease in thrombus weight induced by rt-PA in *in vitro* generated equine thrombi. Interestingly, addition of more than 1.5 μg/ml did not result in a significant increase of thrombolytic activity and thus might be taken as a breakpoint and maximal plasma concentration to be achieved by infusion. Additionally, higher concentrations may increase the risk of hemorrhage without increasing the efficacy. A meta-analysis reveals that treatment of humans suffering from pulmonary thromboembolism with rt-PA is associated with hemorrhage in 8.8% of cases [9].

When comparing the present results with those published for human *in vitro* generated thrombi (although the experimental setting differed slightly), there seems to be a difference in thrombolytic activity between human and equine thrombi. In human thrombi, as little as 150 ng/ml was sufficient to reduce the human thrombus weight by 50% within four hours and a nearly complete lysis of the thrombus was achieved by approximately 600 ng/ml [17]. This difference in efficacy might be due to a slight difference in homology between equine and human t-PA. Two isoforms are described for the equine tissue type plasminogen activator (NCBI protein: accession no. XP_001489324, XP_001489348) and according to BLAST (http://blast.ncbi.nlm.nih.gov/) the homology to the human alteplase is approximately 85%. Thus, equine plasminogen is not the perfect substrate for human rt-PA, which has already been demonstrated in an *in vitro* enzyme activity study [18]. There is approximately a 95% homology for the catalytic side of plasminogen between horse and man (NCBI protein: accession no. AAD25984 and P00747). However, the catalytic activity is less pronounced for human rt-PA [18] and for streptokinase [19]. It is thus speculated that there might be differences in the tertiary structure between human and equine plasminogen [19].

The *in vivo* study complements the finding that rt-PA might be suitable as a thrombolytic agent in horses, as maximal blood concentrations similar to those required for thrombolysis in humans are achieved reliably. By an administration of 1 mg/kg alteplase over a period of 30 min, mean maximal concentrations of 1250 (⋅ 270) ng/ml were reached. This infusion was well tolerated by the horses, and adverse effects were not observed. Only minor changes in blood parameters were noticed in some individuals. The reduced activated partial thromboplastin time at hour 4 observation time might not be of clinical significance as such changes are commonly observed in ill patients or under medication.

This is in accordance with observations in humans. No effects on fibrinogen levels, prothrombin time and thrombin clotting times were observed after infusion of up to 0.5 mg/kg within 30 min in healthy human subjects. An infusion of 0.5 mg/kg resulted in comparable maximal blood concentrations of rt-PA to 1 mg/kg administered to horses in the present study [16]. The clearance rate is similar to findings in other species such as the approximate 10 to 20 mg/ml/min reported in man [6], but the apparent volume of distribution is higher compared to that determined in man [6]. Results obtained in humans showed that the distribution of rt-PA, a protein with a molecular mass of 65,000, is restricted to the intravascular space and well perfused tissues. It is assumed that most rt-PA is removed from the circulation by specific uptake and degradation in the liver [16]. The higher volume of distribution found in horses may be explained by the comparatively long terminal half life. Nevertheless, the concentrations found between 90 min and 300 min are unlikely to play a clinically relevant role.

There are established methods for therapy of thrombi in horses. For example, the coagulation inhibiting potential of heparin is due to accelerating the complex formation between antithrombin and various activated coagulation factors, among which factor Xa and thrombin are the most pivotal ones leading to a prevention of further clot formation [4,20]. In contrast, recombinant hirudin, a direct thrombin inhibitor acts independently of anti-thrombin activity and also inhibits fibrin bound thrombin [5]. The mechanism of action of plasminogen activator alteplase is advantageous in that it activates the thrombus associated plasminogen to plasmin leading to a direct induction of the fibrinolytic system [9]. With these properties rt-PA is an agent that is suitable for an initial thrombolytic therapy that is indicated in early phases of thrombosis, and should be combined or followed by a heparin therapy to be most effective [7,8]. Due to the initial thrombolytic effect it could be expected that a similar therapeutic regimen in horses is superior to commonly performed anticoagulative therapies.

Recombinant t-PA has been used in dogs and cats with varying results. The dosing regimen differs from study to study (reviewed in [9]). Cats received total doses of 1 to 10 mg/kg with variable success. In one case report, a dog with acute distal aortic thrombosis received a bolus injection of 1 mg/kg rt-PA every 60 min 10 times in succession. After six days the dog received another 1.1 mg/kg as boluses given 60 min apart, followed by two 0.7 mg/kg boluses the next day. This procedure led to significant clinical improvement [9].

Results obtained by the *in vitro* thrombolysis indicate a thrombus reduction by approximately 86% when thrombi are incubated with 1.5 μg/ml rt-PA. Based on the *in vitro* findings it seems that at least 1 mg/kg alteplase should be administered to achieve maximal concentrations that lead to optimal lysis of the thrombi.

Collectively the results provide a basis for future therapeutic investigations of thrombotic diseases using rt-PA. The most promising results are anticipated for the treatment of acute jugular thrombi as rt-PA might induce complete thrombolysis. For chronic thrombi (e.g. in the hind limbs) the success might be more limited if results from human patients can be transferred to the horse [21]. In one case report a foal with aorto-iliac thrombosis received a 2 mg dose of rt-PA intravenously. The foal developed a severe coagulopathy and was euthanized [17]. However, considering the pharmacokinetic data obtained in the present study, a 2 mg infusion of rt-PA might be too little to show any efficacy.

## Conclusion

From the *in vitro* and *in vivo* data it is suggested that rt-PA applied via intravenous infusion over a period of 30 minutes at a dose of 1 mg/kg might be suitable for the treatment of thrombotic diseases in horses. This dose regimen was not associated with severe adverse effects which would have led to limitations for the use of rt-PA in thrombotic horses.

## Abbreviations

AUC: Area under the (plasma concentration) curve; CL: Plasma clearance; Cmax: Maximum plasma concentration; t1/2: Plasma half life; rt-PA: Recombinant tissue plasminogen activator.p

## Competing interests

The authors declare no financial or non-financial competing interests.

## Authors’ contributions

WB: performed determination of t-PA in plasma, performed pharmacokinetic analysis and interpretation of data, wrote most of the manuscript. GH performed most of the experiments, was involved in data analysis and interpretation. KF designed study, supervised GH during her experiments and interpretation of results, wrote part of the manuscript. All authors read and approved the manuscript.
